# Active microbiome structure and its association with environmental factors and viruses at different aquatic sites of a high‐altitude wetland

**DOI:** 10.1002/mbo3.667

**Published:** 2018-07-30

**Authors:** Yoanna Eissler, María‐Jesús Gálvez, Cristina Dorador, Martha Hengst, Verónica Molina

**Affiliations:** ^1^ Facultad de Ciencias Centro de Investigación y Gestión de Recursos Naturales Instituto de Química y Bioquímica Universidad de Valparaíso Valparaíso Chile; ^2^ Programa de Biodiversidad and Departamento de Biología Facultad de Ciencias Naturales y Exactas, Observatorio de Ecología Microbiana Universidad de Playa Ancha Valparaíso Chile; ^3^ Laboratorio de Complejidad Microbiana y Ecología Funcional Departamento de Biotecnología Facultad de Ciencias del Mar y Recursos Biológicos Universidad de Antofagasta Antofagasta Chile; ^4^ Centre for Biotechnology and Bioengineering Santiago Chile; ^5^ Departamento de Ciencias Farmacéuticas Facultad de Ciencias Universidad Católica del Norte Antofagasta Chile

**Keywords:** 16S rRNA subunit, archaea, bacteria, high‐altitude wetland, picoplankton, virus

## Abstract

Salar de Huasco is a high‐altitude wetland characterized by a highly diverse microbial life adapted to extreme climatic and environmental conditions. Our study aims to determine active microbial community structure changes within different aquatic sites and its relationship with environmental factors and viruses as potential drivers of diversification in different aquatic areas of this ecosystem. In this study, bacteria and archaea composition (16S rRNA subunit pyrolibraries) and picoplankton and viral abundance were determined at ponds, springs and lagoon sites of the wetland during wet and dry seasons (February and July 2012, respectively). In general, mixosaline waters (1,400–51,000 μS/cm) usually found in ponds and lagoon presented higher picoplanktonic abundances compared to freshwater (<800 μS/cm) spring sites, ranging from 1.07 × 10^5^ to 1.83 × 10^7^ cells/ml. Viral abundance and viral to picoplankton ratio (VPR) also presented greater values at ponds compared to spring sites, reaching up to 4.78 × 10^8^ viruses‐like particles and up to 351 for VPR. In general, ponds hold a higher microbial diversity and complexity associated also with the presence of microbial mats compared with water sources or lagoon (Shannon index H′ 2.6–3.9 vs. <2.0). A greater richness of archaea was also detected in ponds characterized by functional groups such as known methanogens and ammonia oxidizers, and uncultured groups. In total, our results indicate that among the different aquatic sites of the wetland, ponds presented a great microbial community diversification associated to a higher top‐down control by viruses which may influence nutrient and greenhouse gases cycling.

## 1 INTRODUCTION

The high‐altitude plateau (>3,000 m a.s.l.) located in the Central Andes region in South America called Altiplano (Dorador, Vila, Witzel, & Imhoff, 2013; Risacher, Alonso, & Salazar, 2003), contains dozens of aquatic environments, such as wetlands, associated with rivers and springs and also saltier lagoons, lakes and salt flats, which were former paleolakes (Albarracín et al., 2015; Risacher et al., 2003). One of these wetlands is Salar de Huasco, located in the Chilean Altiplano (20º18′S, 68º50′W) at 3,800 m a.s.l. which is a protected site by the Ramsar convention since 1996, whose goal is the conservation and wise use of wetlands and their resources. Salar de Huasco is situated in an arid region (average annual precipitation <300 mm/year) characterized by a high daily temperature fluctuation (−15 to 20°C), mainly during dry austral winter period (Aceituno, 1997), extreme irradiation (up to 1,125 W/m^2^) (Molina et al., 2016), and high‐evaporation rates ~1,260 mm/year, (Risacher et al., 2003). The wet season occurs during austral summer (December–February), which presents high cloud coverage and intense precipitation including snowfall (Garreaud, 1999). The above results in the presence of different landscapes that are associated with meteorological variability, which water dynamics include spring as water sources, streams, ponds, a lagoon areas surrounded by peatlands, a main terminal lake, and a salt flat area (Dorador, Vila, Imhoff, & Witzel, 2008). In terms of salinity classification based on inland aquatic ecosystems and conductivity (Cowardin, Carter, Golet, & LaRoe, 1979); the water sources are typically fresh (<800 μS/cm), whereas in the other aquatic evaporitic sites, water varied in conductivity (μS/cm) from mixosaline, for example, oligosaline (800–8,000), mesosaline (8,000–30,000), polysaline (30,000–45,000), and eusaline to hypersaline (45,000–60,000 and >60,000, respectively). These latter water presents a chemical composition that consists in sulfate, chloride, calcium, sodium, potassium, and magnesium in different proportions than seawater (Risacher et al., 2003). The diverse and heterogeneous aquatic environments (springs, several ponds, and a main lagoon) of the wetland also present variable biogeochemical conditions including nutrients and dissolved organic matter (Aguilar, Acosta, Dorador, & Sommaruga, 2016; Dorador et al., 2008; Hernández et al., 2016). In total, the environmental high‐altitude wetland conditions were consider as drivers of diversification, generating ecological niches for plankton, benthos, microbial mats, and microbialites (Albarracín et al., 2015), which represent a higher degree of microbial complexity. Former results show high microbial diversity, which in terms of bacterioplankton are dominated by the phylum Bacteroidetes, Proteobacteria, Crenarchaeota, Actinobacteria, among others (e.g., Aguilar et al., 2016; Dorador et al., 2013). Archaea and Cyanobacteria are also important members of the microbial community in Salar de Huasco, with representatives such as Methanobacteria and Oscillatoriales, Chroococcales, and Nostocales, respectively (Dorador, Vila, Remonsellez, Imhoff, & Witzel, 2010; Dorador et al., 2008).

High‐altitude wetland microbial communities have been considered as a hotspot of microbial life (Albarracín et al., 2015; Dorador et al., 2013); however, scarce information is available concerning the factors potentially driving this diversity. Limiting environmental factors, such as, temperature, pH, and mineral composition could favor the presence of certain groups of microorganisms, such as endospore‐forming firmicutes in extreme environments (Filippidou et al., 2016). Moreover, factors like salinity could generate niche partitioning among functional groups (Oren, 2001) and salinity plus nutrients were suggested to limit different biotic components of high altitude aquatic environments such as, lakes and lagoons (Márquez‐García et al., 2009). Extreme solar radiation intensity and quality (UV radiation) was found to induce shifts at different levels, from specific cellular response mechanisms (Pérez et al., 2017), up to bacteria community composition level, potentially favoring low abundant and rare groups (<0.5% of orders) able to cope with high UV radiation (Molina et al., 2016).

Overall, environmental factors such as temperature, nature of organic substrates, and nutrients (Tilman, 1977) are denominated bottom‐up controllers, which are important regulators of microbial community size and diversity. However, other source of diversification in aquatic ecosystems includes biological factors such as viral lysis (Larsen et al., 2004) or protistan grazing (Jürgens & Matz, 2002), known as “top‐down control.” So far, little is known about top‐down controllers, such as viruses, which are also expected to affect the active microbial structure in extreme ecosystems such as high‐altitude wetlands. Bottom‐up and top‐down ecosystem forcings were found to act in concert affecting microbial activities of dominant groups, and thus, influence the active community structure in aquatic environments (Pradeep Ram, Chaibi‐Slouma, Keshri, Colombet, & Sime‐Ngando, 2016; Steffen et al., 2015). Moreover, bottom‐up and top‐down processes could generate variable effects within different biotic components of the aquatic food‐web under global warming scenarios, changing the net primary production of the system (e.g., Shurin, Clasen, Greig, Kratina, & Thompson, 2012).

In this paper, we examine the composition of the active microbial community present in different aquatic environments of Salar de Huasco such as spring, ponds, and the main lagoon including sites with higher microbial complexity like ponds with microbial mats, during different periods (wet and dry season) using pyrosequencing of 16S rRNA, to recover most of the variability in this ecosystem. This work describes the active microbial communities at these locations and aims to determine in which aquatic sites there is a greater influence of bottom‐up versus top‐down (viral interaction) on the community structure.

## 2 MATERIALS AND METHODS

**Figure 1:**
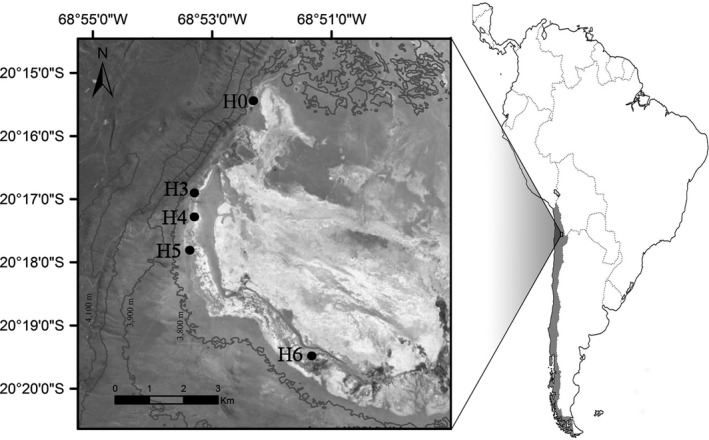
Map of the study area showing the sampling sites for both periods

Sampling took place in Salar de Huasco, Chile, during February and July of 2012, corresponding to wet and dry season, respectively, to cover different sources of variability in the aquatic ecosystems explored, associated with climatic conditions. Samples for picoplankton and viral abundance and for RNA sequencing were taken in 5 (February) and 4 (July) aquatic sites (Figure 1) with different conductivity, including; springs, characterized usually by low conductivity associated with freshwater from groundwater origin; the main lagoon or lake with mixosaline and sometimes hypersaline water and small ponds presenting a wide range of conductivity associated with mixosaline conditions, sometimes having microbial mats (Figures S1 and S2). Depending on their water source, ponds could be transient and disappear associated with desiccation (Hernández et al., 2016). In our case, we only visit the pond at H4 during both dates (Table S1). Several pond sites (i.e., H0, H4, H5 February and H4, H6 July) were located over bacterial mats and sediments showing evident organic matter degradation probably associated with former microbial mats (brown and black color). H0 pond site was sampled 3 and 5 times during February for picoplankton and viral abundance, respectively. Physical and chemical parameters were measured in all sites except for H3 pond in February.

### 2.1 Physical and chemical characterization

Water temperature, conductivity, pH, and oxygen were measured in situ using sensors. Water used for ammonium concentration was collected in triplicate directly in Duran Schott flasks and immediately incubated following fluorometric methods (Holmes, Aminot, Kérouel, Hooker, & Peterson, 1999). Samples were measured using a Turner design^®^ fluorometer. For NO_3_^−^, NO_2_^−^, and PO_4_^3−^ analysis, 60 ml of water were filtered through a 0.45 μm pore (GF/F, Millipore filter) and stored frozen until analyses in a Seal segmented flow analytical AutoAnalyzer AA3, following standard colorimetric techniques (Laboratorio de Biogeoquímica, Universidad de Concepción, Chile).

### 2.2 Viral and picoplankton abundance

Water samples of 15 ml were collected, prefiltered (3 μm, Isopore membrane filter, Millipore) and fixed with glutaraldehyde (1% final concentration), and stored frozen until analyses. Viral samples were first prefiltered (0.2 μm, polycarbonate membrane filter, Millipore) and then processed by filtration onto a 25 mm, 0.02 μm pore‐size Anodisc membrane filters (Whatman) at 130 mm Hg pressure. Filtration volumes ranged between 10 and 1,000 μl (dilution from 1:100 to 1:1). Filtered samples were placed in the bottom of a Petri dish in darkness and stained during 15 min, with 100 μl of the SYBR Gold (10,000X; Invitrogen) that was diluted to a final concentration of 2× in filtered 0.02 μm TE buffer (10 mM Tris and 1 mM EDTA, pH 7.5), according to the method of (Chen, Lu, Binder, Liu, & Hodson, 2001). Filters were then blotted on a Kimwipe (Kimtech^science^) to remove excess fluid, mounted between a glass slide and coverslip. Ten to twenty fields were randomly selected to take photos and count at least 100 viruses per slide at 1,000× using epifluorescence microscopy (EFM). Filters were examined under an Olympus BX60F‐3 epifluorescence microscope, equipped with a HBO 50 W mercury lamp (excitation wavelength 460–490 nm, cutoff filter 515 nm) and a MShot CO‐90 camera. Digital images were processed using Adobe Photoshop CS2 version 9.0 and Image Pro Plus version 4.5.

Water samples of 1 ml were collected using a sterile syringe and fixed with glutaraldehyde (0.1% final concentration) and stored frozen in liquid nitrogen until analysis. Subsamples (150 μl) were analyzed with a FACSCalibur flow cytometer equipped with an ion–argon laser (488 nm of 15 mW, Becton Dickinson). The abundance of nonautofluorescent picoplankton was estimated from separate samples previously stained with SYBR‐Green I (10,000X; Molecular Probes) (Marie, Partensky, Jacquet, & Vaulot, 1997). DNA was stained with SYBR‐Green I before estimating the total picoplankton abundance through flow cytometry (FCM). In addition, picoplankton abundance was also estimated for four samples using EFM following the same protocol described above for viral abundance but using a 0.2 μm pore‐size Anodisc membrane filter (Whatman). Picoplankton counts obtained through FCM were used for further statistical analyses and estimations, depending on data availability.

### 2.3 RNA extraction and treatments

Water samples of 60 ml were filtered in situ through a 0.22 μm pore‐size filter (GVWP02500, Millipore, 25 mm diameter) using a sterile syringe and filter holders. RNAlater^®^ solution (Ambion, Life Technologies, USA) was added before storing the filter in liquid nitrogen in the field, and transferred to −20°C until further analyses.

RNA was extracted using the mirVana miRNA Isolation Kit (Ambion, Life Technologies) according to the manufacturer's protocol. A mechanical disruption phase was included using 200‐μm‐diameter zirconium beads (Low Binding Zirconium Beads, OPS Diagnostics, Lebanon, NJ) and then homogenized twice for 30 s using a Mini‐Beadbeater‐8^™^ (Biospec Products, Bartlesville, OK) to ensure complete cellular disruption.

Quantification and quality of RNA was determined spectrophotometrically (Synergy Mx Microplate Reader, BioTek Instruments) before using Turbo DNA‐free kit (Ambion, Life Technologies) for DNase treatment and reagent removal procedure. The kit ImProm II^™^ Reverse Transcription System (Promega Corp, Madison, WI) was used for generating complementary DNA (cDNA) with the provided random primers.

### 2.4 Pyrosequencing analysis

Bacterial and archaeal 16S rRNA gene pyrolibraries from V1–V3 and V3–V5 region, respectively, were generated from cDNA preparations using the primers 28F (5′‐GAG TTT GAT CNT GGC TCA G‐3′) and 519R (5′‐GTN TTA CNG CGG CKG CTG‐3′) for bacteria and arch349F (5′‐GYG CAS CAG KCG MGA AW‐3′) and arch958R (5′‐YCC GGC GTT GAM TCC AAT T‐3′) for archaea at the Research and Testing Laboratory (RTL, TX).

16S rRNA gene sequences were curated following the Ribosomal Data Project pipeline by removing primers and barcodes, filtering low quality and length reads, including sequences with ambiguity codes (Cole et al., 2014). The trimmed sequences were then taxonomically classified using the automatic software pipeline SILVAngs (v128) available from https://www.arb-silva.de/ (Quast et al., 2013). The 16S rRNA pyrolibraries were deposited in the European Nucleotide Archive under study accession PRJEB21175 and the following specific accession runs ERR1999886–ERR1999903.

### 2.5 Microbial community and statistical analysis

Analyses were performed using R 3.1.3 statistical software and different available packages, unless stated otherwise.

A Spearman rank correlation was performed to understand the relationship between environmental parameters and microbial abundance using Statistica version 7.0 software.

Single sequences obtained from 16S rRNA pyrosequencing were removed from further analysis to avoid bias. Rarefaction curves were created using Jenna Jacobs' function (http://www.jennajacobs.org/R/rarefaction.txt). Microbial community was analyzed using operational taxonomic units (OTU), considering 93% of similarity by estimating the Shannon (H′) index for diversity, Pielou's evenness, and Fisher's alpha index using the “Vegan package” (Oksanen et al., 2016).

A heatmap and cluster analysis was done using the phyla taxonomic level, for which data was first normalized using the fourth‐root‐transformation and the columns (sites) were then z‐score scaled. Cluster analysis was performed using “Pvclust package” based on Euclidean distance and Ward.2 method. The approximately unbiased was used as *p*‐value, which also computes a limited number of bootstrap samples, with an *α* = 0.95. The heatmap was based on Manhattan distance using the Heatmap.2 function from the “Gplots package.”

In order to visualize the active microbial community structure changes between the different sites and periods studied a multidimensional scaling (MDS) and principal coordinates analysis (PCoA) were estimated. These analyses were based on a Bray Curtis similarity matrix of square‐root transformed data of the active bacterial phyla using the software PRIMER (7.0.11) with the PERMANOVA add on (Anderson, Gorley, & Clarke, 2008). Correlation analyses (Pearson) were estimated and overly was done in both analyses to visualize the main phyla contribution to the similarity distribution at the different sites and periods studies (MDS) and the environmental variables (precipitation, conductivity, oxygen, pH, NO_3_^−^, NO_2_^−^, PO_4_^3−^, and NH_4_^+^) in the case of the PCoA. Similarity Percentage Analyses (SIMPER) were carried out to determine the contribution of active phyla or domain to the similarity or dissimilarity associated with specific discriminatory factors selected based on PCoA and arbitrary determinants such as viral abundance.

## 3 RESULTS

### 3.1 Environmental characteristics and microbial abundance

**Figure 2:**
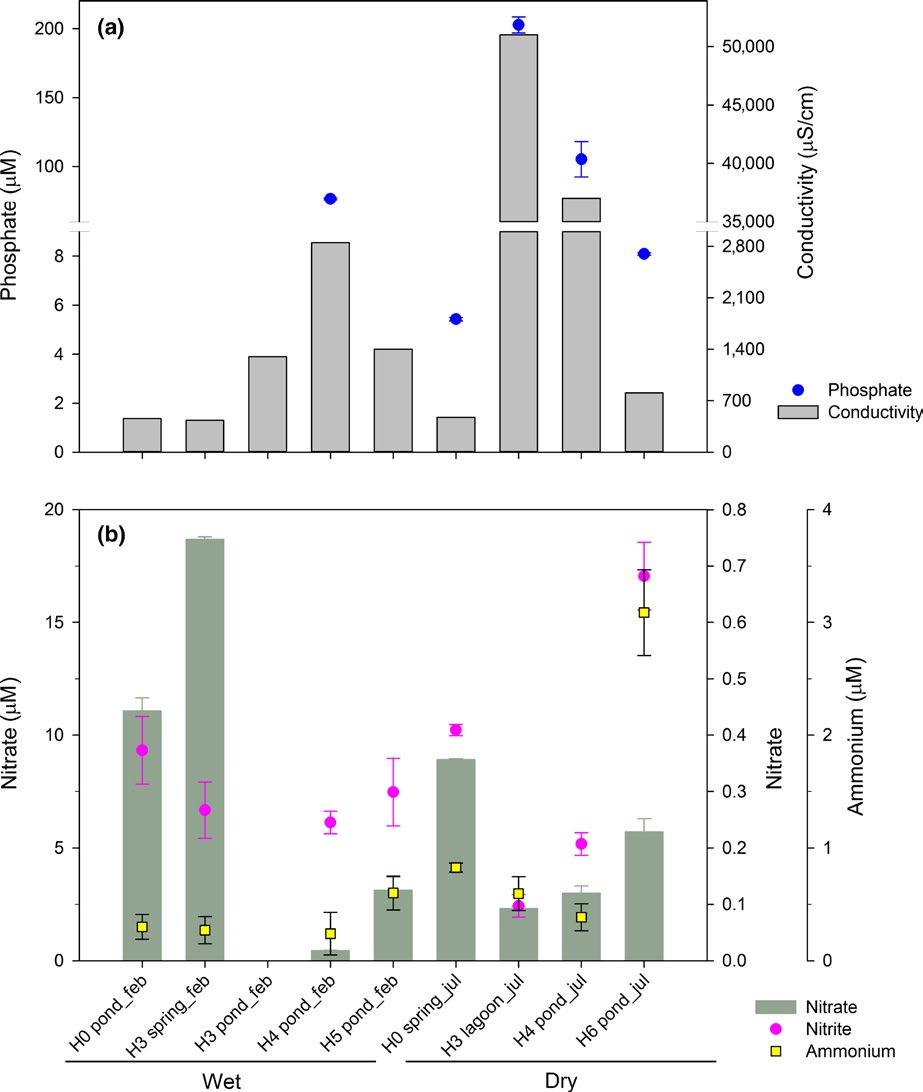
(a) Conductivity and phosphate concentration and (b) nitrate, nitrite and ammonium concentration changes associated with the different sampling sites during contrasting seasons (wet vs. dry)

Physicochemical parameters of water sampled are shown in Table S1. The dry and wet periods were characterized by average monthly precipitation of 0 (July, austral winter season) and 75.2 mm (February, austral summer season), respectively. This contrast was qualitatively observed in the water level of the main lagoon and at ponds sites. Also, water temperature ranged from 5.1 to 22.5°C at different sites during sampling time, with higher values registered during the wet season, whereas lower temperature values were measured during the dry season (Table S1). Besides, during dry season at dawn the water at ponds was frozen before sampling took place (Figure S2). Conductivity (Figure 2a) ranged between 434 and 51,000 μS/cm, the higher values were registered in H3 lagoon and H4 pond during dry season and lowest at H3 spring, H0 pond sites during wet season and H0 spring, dry season. Conductivity values <806, 1,300–2,850 and >37,000 μS/cm indicate different types of water surveyed during this study as freshwater and mixosaline such as oligosaline and close to mesosaline conditions according to Cowardin et al. (1979).

Also, the pond sites presented a higher variability of oxygen and pH (4 to 8.5 mg/L and pH from 6.3 to 9.3, Table S1), whereas the spring sites presented more stable oxygen and pH conditions, regardless the season.

Nitrite varied from 0.10 ± 0.02 to 0.68 ± 0.06 μmol/L, with both values observed during dry season (H3 lagoon, H6 pond, respectively). Ammonium showed the highest concentration (3.09 ± 0.38 μmol/L) for H6 pond during dry season and the lowest (0.24 ± 0.19 μmol/L, H4 pond) during wet season. Nitrate, presented the lowest (0.45 ± 0.01 μmol/L, H4 pond) and the highest (18.69 ± 0.11 μmol/L, H3 spring) concentration during wet season (Figure 2b). Phosphate concentration ranged from 0.60 ± 0.04 μmol/L in H3 Spring (wet season) to 202.69 ± 5.84 μmol/L in H3 lagoon (dry season) (Table S1 and Figure 2a).

Significant Spearman rank correlations among environmental variables, were only found between conductivity and PO_4_^3−^ (*r* = 0.93) and NO_3_^−^ (r = ‐0.93) (both *p *<* *0.001), and NO_3_^−^ and PO_4_^3−^ (*r* = −0.86, *p *<* *0.01).

**Figure 3:**
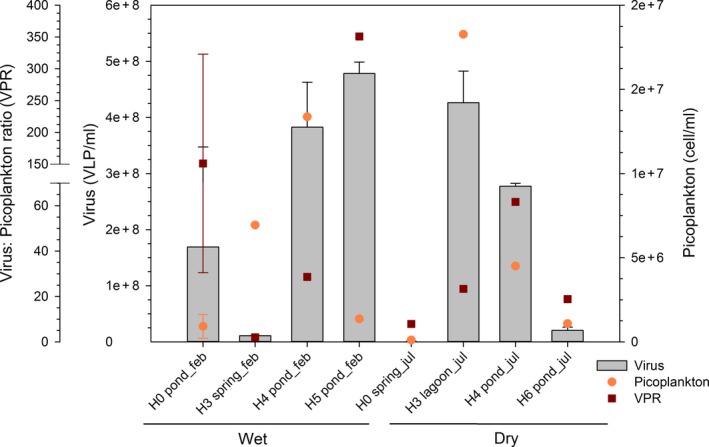
Picoplankton and viral abundance, determined by FC and epifluorescence microscopy, respectively and viruses to picoplankton ratios associated with the different sampling sites during wet and dry periods

There was a strong variation in picoplankton and viral abundances during this study. Picoplankton abundance estimated through FCM ranged between 1.00 × 10^5^ and 1.83 × 10^7^ cells/ml in H0 pond and H3 lagoon during wet and dry season, respectively (Figure 3 and Table S2). Picoplankton abundance determined with EFM were only available for four samplings during wet season and ranged from 5.55 × 10^4^ ± 7.16 × 10^3^ (H0 pond) to 3.27 × 10^6^ ± 1.14 × 10^5^ (H3 spring) cells/ml (Table S2). Comparing EFM with FCM results, the first technique showed lower values, from more than one order of magnitude (H0 pond, 5.55 × 10^4^ vs. 1.33 × 10^6^ cells/ml) to almost the same value at H5 pond (Table S2). In addition, when samples were analyzed with EFM showed organic matter aggregates (Figure S3). Viral abundance varied from 8.44 × 10^5^ ± 5.09 × 10^5^ (H0 spring, dry season) to 4.78 × 10^8^ viruses‐like particles (VLP) per ml ± 2.05 × 10^7^ (H5 pond, wet season) (Figure 3 and Table S2). High values of viral abundance (>1 × 10^8^ VLP/ml) were observed at aquatic sites with greater conductivity values, such as H4 and H5 pond sites during wet season and at H3 lagoon and H4 pond sites during dry season, except for H0 pond freshwater site. The virus (derived from EFM) to picoplankton (based on FCM) ratios (VPR) ranged from 2 (H3 spring) to 351 (H5 pond), both from wet season (Figure 3). These values were higher when calculated using viral and picoplankton abundance determined by EFM for the sites whose data were available (Table S2). Pond sites presented viral abundance higher than 10^8^ VLP/ml up to three orders of magnitude compared to spring areas (Table S2). Besides, pond sites presented microbial mats mainly during wet season, when mats were more conspicuous and widespread (Figures S1 and S2). Picoplankton abundance (FCM) was only correlated to NO_2_^−^ (*r* = −0.83, *p *<* *0.01) and viral abundance (EFM) to pH (*r *= 0.76) and NO_3_^−^ (*r* = −0.74), both presented *p *<* *0.05 (Table S3).

### 3.2 Active microbial community composition comparison between sites

**Figure 4:**
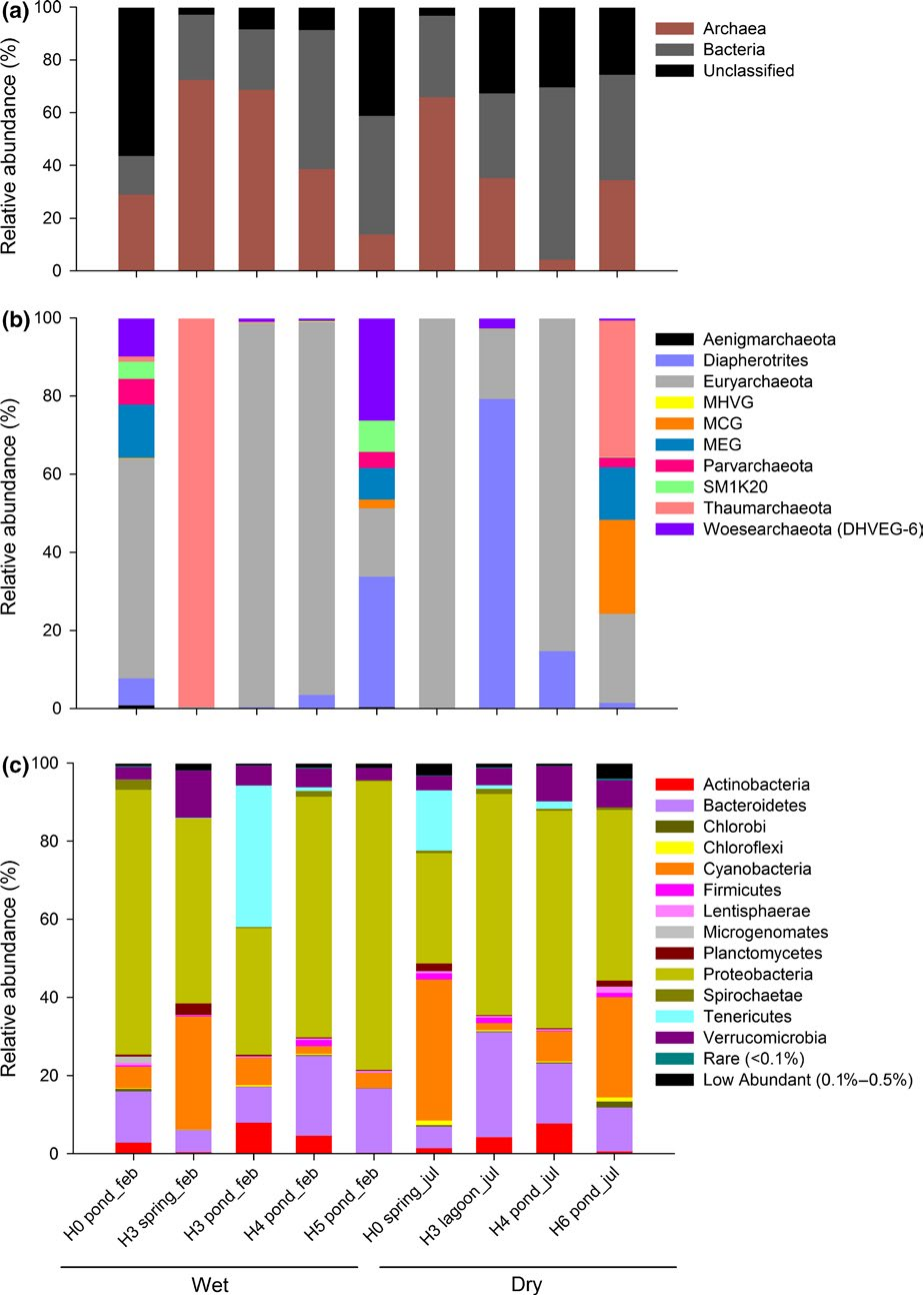
(a) Relative contribution of classified domains based on pyrosequencing 16S rRNA subunit and the proportion of unclassified sequences in databases. (b,c) Archaeal and bacterial phyla relative contribution, respectively, retrieved at the different sampling sites during contrasting seasons (wet vs. dry)

Between 5,218 and 34,575 sequences were obtained per sample, of which 40.4% to 92.6% corresponded to assigned sequences (Table 1). Archaea and bacteria represented between 4.2% to 72.5% and 14.7% to 65.0%, of the total assigned sequences (Figure 4), respectively and were associated with 3–42 and 133–338 OTUs, respectively. Mixosaline ponds and lagoon presented a higher number (25.6%–56.4%) of unclassified sequences (Figure 4a).

**Table 1:** 16S rRNA subunit sequencing results, including richness and Alpha diversity results

	Total sequences (no.)	Sequences assigned (%)	Operational taxonomic units (no.)	Chao	Shannon (log1)	Evenness (Pielou's)	Fisher (a)
H0 pond_feb	11,702	40.9	342	506	2.97	0.54	47.4
H3 spring_feb	29,284	90.3	311	408	2.00	0.36	41.7
H3 pond_feb	7,237	69.5	184	292	2.63	0.50	33.0
H4 pond_feb	10,268	48.9	247	333	3.39	0.64	40.4
H5 pond_feb	5,218	51.2	274	520	3.00	0.56	44.2
H0 spring_jul	34,575	92.6	379	433	1.73	0.30	51.7
H3 lagoon_jul	14,166	51.9	226	324	1.77	0.36	22.4
H4 pond_jul	6,573	40.4	283	477	3.59	0.66	51.2
H6 pond_jul	12,303	64.2	591	786	3.88	0.64	96.3

The rarefaction curves (Figure S4) indicated that only three sites (i.e., H3 spring during wet season, H0 spring and H3 lagoon, during dry season) out of nine reached a plateau, suggesting that not all the diversity was retrieved from most of the sites and particularly from ponds. Moreover, the highest alpha diversity indexes (i.e., Shannon index) were determined at pond sites, particularly during dry season, compared with spring and lagoon (Table 1).

A total of 52 phyla were observed among archaea and bacteria, including rare and low abundant phyla considering the different sites (Figure 4b,c). A total of 10 archaeal phyla were detected, but only Euryarchaeota followed by Diapherotrites, Thaumarchaeota, and Woesearchaeota were predominant (Figure 4b). The sites with high archaea richness including uncultured phyla were mostly detected at pond sites independently from the season (i.e., H0, H5, and H6), whereas Diapherotrites reached high abundance during dry season, particularly at H3 lagoon. Ponds presented a higher richness of many orders associated with functional groups, such as known methanogens and ammonia oxidizers (Figure S5a). Moreover, the order Methanosarcinales represented more than 50% at H0 pond where also microbial mats were developed. This order was dominant at the spring site H0 during dry period. Thermoplasmatales, including also methanogens, were highly dominant at H3 pond (98%) during wet season and well represented at H4 pond (46 and 38%) during both seasons.

A total of 42 bacterial phyla were retrieved, characterized by the high contribution of Proteobacteria, followed by Bacteroidetes, Cyanobacteria, and Verrucomicrobia, including also low abundant <0.1%–0.5% (*n* = 15) and rare phyla <0.1% (*n* = 13), considering all the study sites (Figure 4c). Among, Proteobacteria phyla, the most abundant classes were Alphaproteobacteria (16%) followed by Gammaproteobacteria (13%) and Betaproteobacteria (11%) considering both sampling periods. However, other Proteobacteria classes reached higher contribution at ponds compared with spring or lagoon, such as Alphaproteobacteria and Betaproteobacteria, that is, H5 and H0 pond sites, 45% and 54%, respectively, during wet season and Epsilonproteobacteria at H6 pond ~9.5% during dry season (Figure S5b). At the lagoon, Cytophagia, Flavobacteriia were predominant classes within Bacteroidetes. Overall Cyanobacteria and Chloroplast classes were widespread, while only Chloroplast predominates at H0 spring (35%) and ponds (7% and 24%) during dry season. In addition, at ponds other classes present a high contribution, for example, Mollicutes and Actinobacteria, among others (Figure S5b).

### 3.3 Active microbial community structure influenced by environmental factors and viruses

The active microbial community structure visualized by cluster and heatmap analysis, indicated the presence of three subclusters, not clearly associated with the seasons, but with aquatic sites, since only one cluster was significantly differentiated and constituted by H0 and H5 pond sites from wet season and H6 pond from dry season (Figure S6).

**Figure 5:**
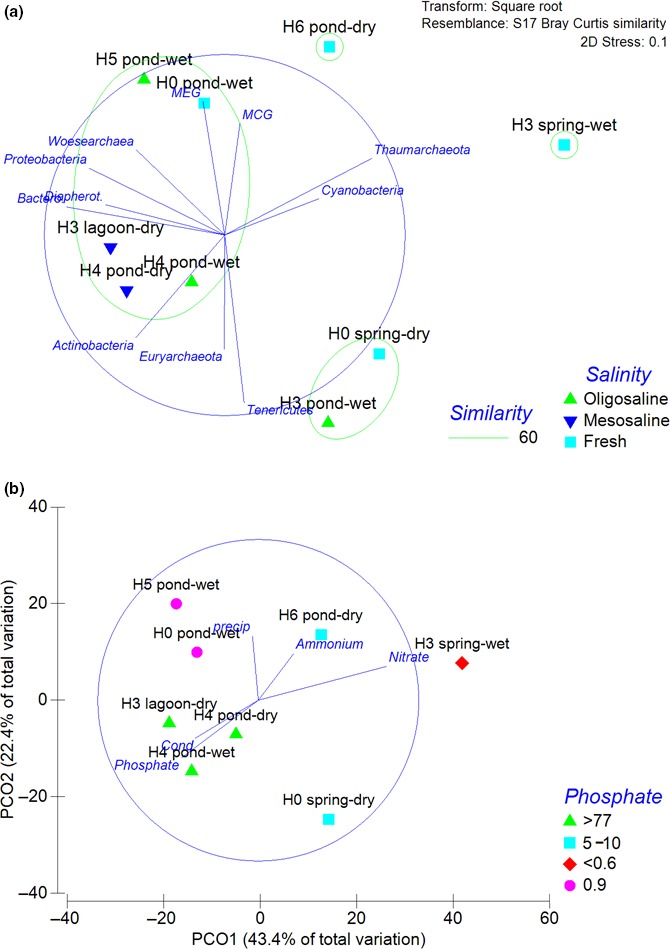
Multidimensional scaling (a) showing the variability of active microbial community structure associated with the different sampling sites in association with salinity based on the following conductivity thresholds (fresh oligosaline and mesosaline <806, 806–2,850, >37,000 μS/cm, respectively). For H3 pond wet season, there is not available nutrients information. Principal coordinates analysis ordination (b) showing environmental factors potentially relevant in the study area. Nitrite and pH were not shown, but overlap with ammonium and conductivity, respectively

MDS and Principal Coordinates Analyses (PCoA) presented in Figure 5a and b, showed the active microbial phyla similarity distribution at the different sampling sites. The microbial community associated mesosaline conditions (>37,000 μS/cm) from H3 lagoon‐dry and H4 pond‐wet presented a higher similarity (68%–79%) and shared ~60% similarity with oligosaline and fresh water sites H5 and H0 pond from wet season. The microbial community associated with the other three sites presented a higher dissimilarity, showing no association with the sampling season. Based on a similarity analysis (SIMPER), considering conductivity as a discriminatory factor, the similarity between sites was explained by the contribution of Proteobacteria >22% in all sites, followed by Bacteroidetes (>16%) at mixosaline sites and Euryarchaeota at fresh water (>17%). In addition, this analysis, indicated that Archaea was the most relevant differentiated group among sites. The phyla Euryarchaeota and Diapherotrites contributed with 35.7% to the dissimilarity between the following discriminatory factors between mesosaline and oligosaline conditions, whereas Thaumarchaeota and Euryarchaeota with 26.6% between fresh and oligosaline sites. PCoA ordination analyses presented as expected a similar distribution of the similarity as MDS. The vectors overlying PCoA analyses (Figure 5b) showed a negative correlation of conductivity, pH and phosphate (*r* > −0.4) and a positive correlation of nitrite and nitrate (*r* = 0.8 and 0.4, respectively) with Axis 1 (43.4% of total variation), whereas precipitation (*r* = 0.4) presented a correlation with Axis 2 (22.4% of total variation).

**Figure 6:**
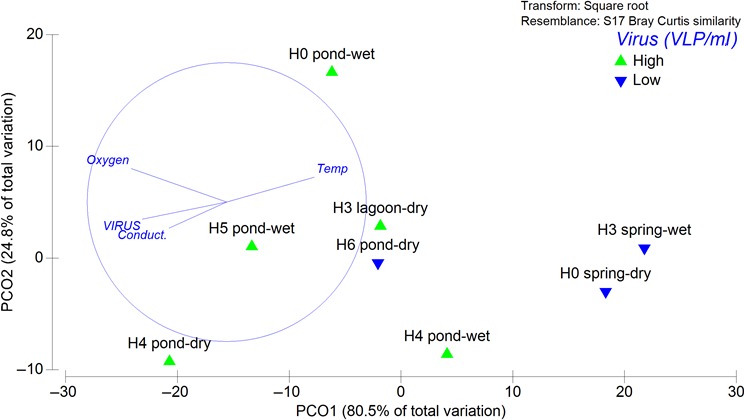
Principal coordinates analysis ordination showing the variation in archaeal and bacterial domain and the proportion of unclassified sequences from 16S rRNA subunit libraries at the different sites

PCoA ordination analysis based on domain contribution (archaea and bacteria), including unclassified sequences based on the current database at the different sampling sites are presented in Figure 6. The most similar community was associated to spring sites during both sample periods, to the H3 lagoon, H6 pond during dry period, whereas a higher dissimilarity was observed mainly at pond sites during wet period. The vectors overlying PCoA analyses (Figure 6) showed a negative correlation for oxygen, virus and conductivity (*r* = −0.7, −0.5, −0.4, respectively) and a positive correlation for temperature (*r* = 0.6) with Axis 1 (80.5% of total variation), whereas virus (*r* = 0.6) presented a correlation with Axis 2 (24.8% of total variation). Based on SIMPER analysis, using an arbitrary discriminatory factor was included based on VLP abundances considering samples characterized by ≥10^8^ VLP/ml as high and <10^8^ VLP/ml as low (Table S4). The grouping analyses indicate that the dissimilarity was explained by the contribution of unclassified groups followed by archaea (41 and 39%, respectively).

## 4 DISCUSSION

### 4.1 Environmental characteristics and microbial abundance in the different aquatic sites studied

Salar de Huasco wetland was characterized by having different ranges of conductivity and nutrients, associated with the aquatic type sampled (springs, ponds, lagoon) independently to the sampling season, dry (July) or wet (February), having a potential effect on microbial distribution including picoplankton and viruses both reaching a high abundance at evaporitic areas and ponds with microbial mats. The wetland presented a high daily variability affecting significantly the water balance in the area specially sites situated far from groundwater supply, such as ponds and lagoon (evaporitic areas) compared with spring sites. In addition, during our study, daily variability was associated with notorious temperature changes, from freezing point at dawn (Figure S2b and e, photographs showing ice coverage) to around 20°C at hours of highest solar radiation. This fluctuation is characteristic of this ecosystem (see meteorological station real‐time data available from 2015 in http://www.ceazamet.cl/index.php?pag=mod_estacion&e_cod=SALH&p_cod=ceazamet). The temperature differences were particularly evident in the water temperature measured in situ during dry and cold seasons, as sampling took place from station H6 toward H0 (Table S1).

Along with physical changes, conductivity and nutrients distribution also varied with the aquatic type sampled. Spring sites were characterized by fresh and high nitrate and low phosphate concentration, compared with the other areas. These characteristics were found to be consistent with our previous data, using similar analytical methods at the spring site H0 (Hernández et al., 2016; Molina et al., 2016). On the other hand, ponds areas were mixosaline and with variable nutrients concentration. Also, conductivity was correlated to nitrate and phosphate, both variables peaked at ponds (and the lagoon) during dry period (Figure 2). The higher phosphate concentration determined at the lagoon and pond sites (5–211 μM) could be related with high organic matter availability due to remineralization at these sites. Indeed, a high benthic primary production has been reported at the lagoon of Salar de Huasco (de la Fuente, 2014). Moreover, a high microbial activity based on ^3^H‐leucine incorporation was determined at ponds in the same study area, from a later sampling carried out at November 2014 (dry season), showing also a significant correlation between the microbial activity and phosphate and conductivity (Hernández et al., 2016). Moreover, during our study, the ponds and the lagoon at the wetland characterized by oligosaline and close to mesosaline conditions hold an abundant picoplankton community compared with spring sites (Figure 3), indicating a higher microbial activity at these sites. Conductivity, was positive but not significantly correlated with picoplanktonic abundance (Table S3) but was the factor that explain most of the variability in microbial community structure in the study area. In total, this result support other study in extreme ecosystems from Antarctica, showing that high salinity aquatic environments present higher picoplanktonic abundances compared to freshwater sites (Laybourn‐Parry, Hofer, & Sommaruga, 2001).

In general, as picoplankton abundance, viral counts were high (>10^8^ VLP/ml) at the oligosaline and mesosaline lagoon and ponds) including the H0 pond during wet period, which was also characterized by having microbial mats (Figure S1). These results partially support reports showing high viral abundance reaching 10^10^ VLP/ml in saline environments. For example, hypersaline solar salterns from Tunisian systems were characterized by the highest viral concentrations reported for any aquatic ecosystem (Boujelben et al., 2012).

On the other hand, microbial mats could also have significant VLP counts, for example, photosynthetic mats from marine intertidal environments, having similar mesosaline conditions than the lagoon, presented a higher number of viruses compared with sediments, ~10^10^ and 10^7^–10^9^ VLP/g, respectively (Carreira, Staal, Middelboe, & Brussaard, 2015). In these shallow aquatic sites, sediments and especially microbial mats could be considered as a potential source of microorganisms including viruses to the overlying water. During our study, microbial mats were observed in ponds sites during both seasons but were more clearly developed at H0 pond during wet season (Figures S1 and S2). In fact, microbial mats besides holding a rich microbial community, are characterized by having high viral abundance, between 9.6 × 10^8^–1.2 × 10^9^ VLP/g (unpublished data, H0 pond site sampled during November 2014). In addition, organic matter aggregates in water samples were observed during our microscopic epifluorescence analyses to be inhabited by a rich microbial community including viruses (Figure S3).

### 4.2 Active microbial community structure and environmental forcing in diverse aquatic ecosystems in the wetland

During our study, the active microbial community composition of the wetland was characterized by changes in the contribution of microbial domains studied (Figure 4), and archaea were identified to contributed greatly to this dissimilarity (Figure 5a). In fact, mixosaline ponds (oligo and mesosaline) presented a higher richness of archaea including the presence of previously reported phyla Euryarchaea described in earlier 16S rRNA gene surveys (e.g., Dorador et al., 2013). Ponds were also characterized as sites with the greatest proportion of unclassified sequences, suggesting a higher microbial novelty. In addition, many of the active microbial communities detected may be related with carbon limit ecosystems or even depending on chemoautotrophy and anaerobic metabolism. Examples associated with these findings are described below.

Among archaea, methanogens and salt tolerant groups such as Halobacteriales were rich at ponds sites during dry period (Figure S5a). Moreover, the order Thermoplasmatales was dominant at H3 pond and shared with Halobacteriales the archaeal contribution at H4 pond site during both wet and dry periods. Thermoplasmatales were mostly associated with CCA47, VC2.1Arc6, and KTK 4A sequences from uncultured archaea associated to extreme ecosystems, for example, deep‐sea methane seeps (Ruff et al., 2013), soda lake (Lanzén et al., 2013), and hydrothermal chimneys (Moussard, Moreira, Cambon‐Bonavita, López‐García, & Jeanthon, 2006), the first one is usually found in marine but also in subsaline ecosystems (Ferrer et al., 2011). Thermoplasmatales and Halobacteriales were described previously at Salar de Huasco using clone libraries (Dorador et al., 2010) and they recently reported to be important groups in salt crusts also from the hyperarid Atacama desert (Finstad et al., 2017). Also, Diapherotrites from the DPANN superphylum (Rinke et al., 2013) peaked at the lagoon site during the dry period, another potential salt tolerant group. This archaeon corresponds to an archaeal novel phylum potentially able to live on few substrates according to genome analyses *Candidatus* “Iainarchaeum andersonii” from a ground water seep gold mine (Youssef et al., 2015). Interestingly, single cell genomics analysis of Diapherotrites revealed the reduced genome size of this group and loss of important anabolic and catabolic pathways (Rinke et al., 2013). On the other hand, other chemoautotrophic groups were detected, the order Nitrosopumilales at H3 spring, which includes functional ammonia oxidizing groups. Among bacteria, a higher contribution of the Mollicutes class bacteria were found at H3 pond‐wet, affiliated to the uncultured group NB1‐n, currently proposed as “*Candidatus Izimaplasma* sp.” (Skennerton et al., 2016). This group was enriched from deep‐sea methane seeps sediments, is the only free‐living bacteria member from the Mollicutes class and its metagenomic analysis suggests that has an anaerobic fermentation metabolism. Another group with potentially the same metabolism (*Opitutus* sp. from the Verrucomicrobia phyla) (van Passel et al., 2011), was found during both sampling periods, predominantly at H3 spring (wet season). Other example of anaerobic and facultative bacteria were the ones from the classes Epsilonproteobacteria and Gammaproteobacteria (Figure S5) associated to the genera *Sulfuricurvum* sp., *Sulfurimonas* sp. and *Rheinheimera* sp., *Thioalkalivibrio* sp., respectively. These genera were reported to be characteristic of sulfur and iron rich environments (Mitchell, Rocha, Kaakoush, O'Rourke, & Queiroz, 2014). In addition, Actinobacteria and Bacteroidetes, well‐known salt tolerant groups, usually present in seawater (e.g., Walsh et al., 2016), were detected at mixosaline areas of the wetland (ponds and lagoon), together with moderate halophilic nitrogen fixers Cyanobacteria, such as, *Nostoc* and *Oscillatoria* (e.g., Zhang & Feng, 2008).

A potential explanation of the active microbial phyla variability was associated with environmental conditions, such as conductivity and nutrients, suggesting a potential bottom‐up control of microbial community structure particularly at the ponds (Figure 5a,b). Dissolved organic matter was found to be significant at pond sites in a previous study carried out at site H3 ponds (Aguilar et al., 2016). In addition, salinity is a stress factor associated with the microbial community metabolism (Oren, 2001). For example, studies in estuaries indicate shifts within functional groups related with archaea such as methanogens and ammonia oxidizers, the first favored at freshwater and the second at higher salinity areas (Xie, Zhang, Zhou, & Wang, 2014).

### 4.3 Interaction between viruses and picoplanktonic communities

Salar de Huasco has been described as a polyextreme ecosystem and thus multiple factors could explain microbial diversification and community composition variability. In one way, microbial community composition can be linked to bottom‐up control by nutrient availability and conductivity as important forcing factors of microbial community structuring as described for many microorganisms according to their tolerance and metabolisms (Oren, 2001; Xie et al., 2014). On the other hand, viruses were also associated with the spatial distribution of microbial domains along with physical and chemical variables such as oxygen, conductivity and temperature (Figure 6). During our study, VPR values ranged from 2 to 351, reaching the highest values specially at pond sites during wet season, that is, H0 (VPR = 151 ± 172) and H5 (VPR = 351) and the lowest at spring sites (both seasons). The VPRs were different considering the techniques reported here, showing a higher value when virus and picoplankton abundance were determined using EFM compared with FCM for picoplankton (Table S2). Picoplankton fraction could be underestimated during EFM counts in the study area considering the presence of microbial aggregates (Figure S3), as suggested by (Luef, Neu, & Peduzzi, 2009), resulting in higher VPRs. Furthermore, EFM could overestimate viral abundance since DNA associated with membrane‐derived vesicles, gene transfer agents, or cell debris can produce fluorescent dots that can be confused with viral particles (Forterre, Soler, Krupovic, Marguet, & Ackermann, 2013). Future studies in the area should consider the need of take more replicates and the comparison of different techniques, for example, VLP determination using FCM. In spite of these potential uncertainties or biases in the abundance determinations, VPRs were in general close but higher than previous results observed in natural environments (Wommack & Colwell, 2000). Usually, VPRs in aquatic systems falls between 3 and 10, reaching maxima at more productive environments (extensively discussed in Wommack & Colwell, 2000; Weinbauer, 2004). For example, at freshwater and saline lakes, this ratio has been found to be as high as 76 and 50, respectively (Laybourn‐Parry et al., 2001; Wommack & Colwell, 2000) and in Antarctic lakes as high as 141 (Kepner, Wharton, & Suttle, 1998). At the study area, a higher microbial activity based on picoplankton abundance and richness was detected at ponds, including sites with higher VPR, potentially related with a higher productivity, particularly at sediments with microbial mats.

VPR are indicative of viral–host interaction through a lytic activity (Weinbauer, 2004; Wommack & Colwell, 2000); therefore, the high ratios found here suggest a great host–virus interaction via microbial cell lysis in this extreme ecosystem. However, further studies are necessary to establish the extent of this interaction, which for instance should include viral lysis versus flagellate grazing (e.g., Allen, Willner, Oechel, & Lipson, 2010; Ram et al., 2013), which would help to understand how nonselective grazing and host specific viral lysis differentially affect microbial community structure. In according to the model “Killing the Winner” microbial richness and evenness are top‐down controlled by viral lysis and microbial community composition is bottom‐up controlled through host nutrient competition (Thingstad, Vage, Storesund, Sandaa, & Giske, 2014). Consequently, viruses could play an important role in the diversification of the microbial communities and help to explain why this high‐altitude wetland harbors a hotspot of microbial diversity.

## 5 CONCLUSIONS

The high‐altitude wetland of Salar de Huasco presents a variety of aquatic environments that is, springs, ponds, and the lagoon, characterized by a rich, diverse and active microbial community. Among these sites, mixosaline ponds were characterized by higher microbial diversity and richness of functional archaeal groups associated with methanogenesis and ammonia oxidation compared with water sources spring and terminal lake. In addition, ponds, were areas of high microbial abundance including high VLP and significant VPRs, with the presence of mats and sediments that could be a reservoir and source of microorganisms to the water column. Ponds could be considered as diverse reservoirs of microbial life characteristic of high‐altitude wetlands.

## CONFLICT OF INTEREST

The authors declare no conflict of interest.
