# Supplementary material for: Active microbiome structure and its association with environmental factors and viruses at different aquatic sites of a high‐altitude wetland
Source: Microbiologyopen. 2018 Jul 30;8(3):e00667. doi: 10.1002/mbo3.667 (PMC6436485; doi:10.1002/mbo3.667)
Supplement: Supplementary file 1 [file MBO3-8-e00667-s001.docx]

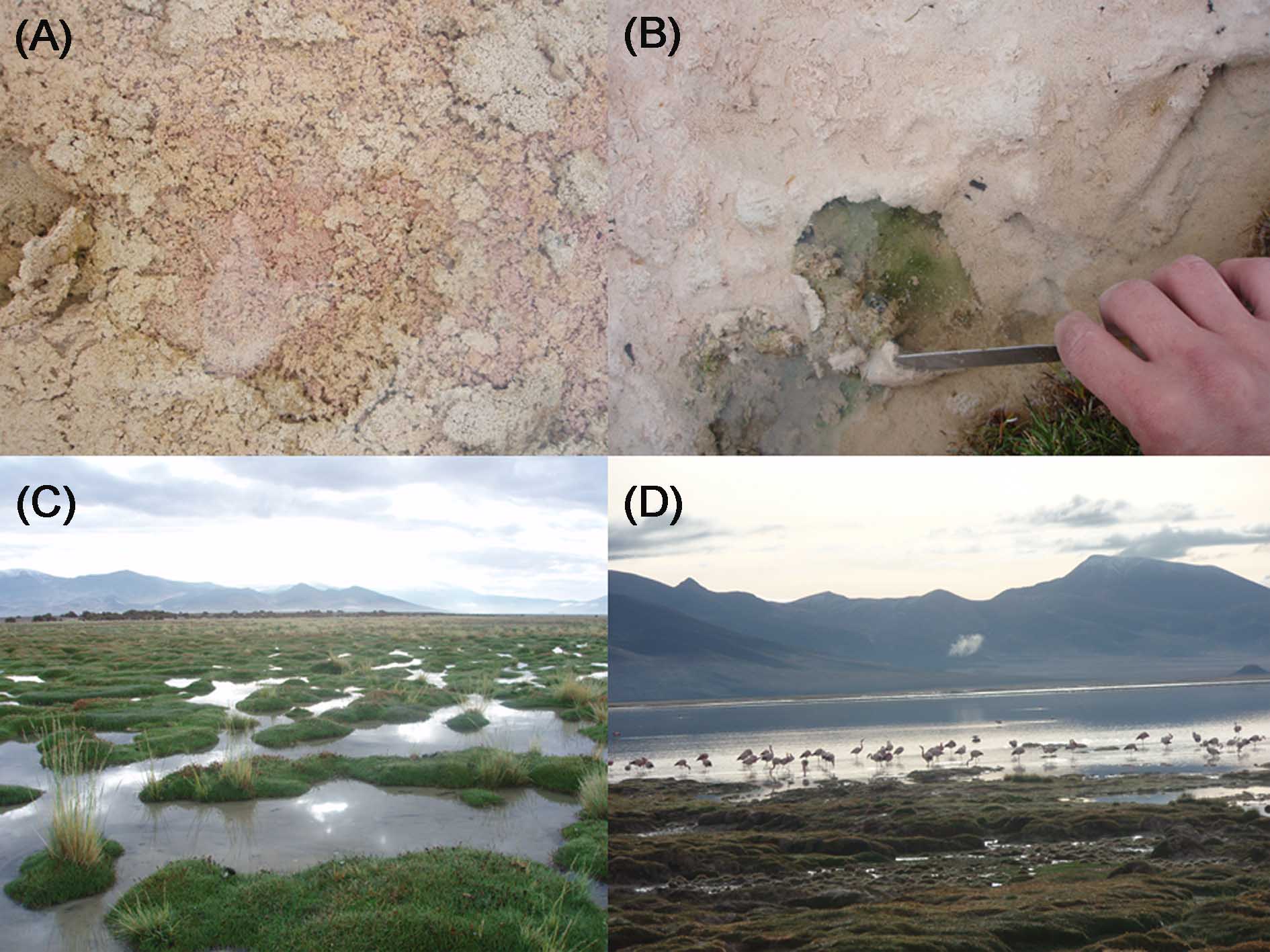


Figure S1. Sampling sites images for February 2012 (wet season). (A) and (B) H0 and H4 pond sites, respectively showing microbial mats, (C) and (D) H3 landscapes, ponds and lagoon, respectively.


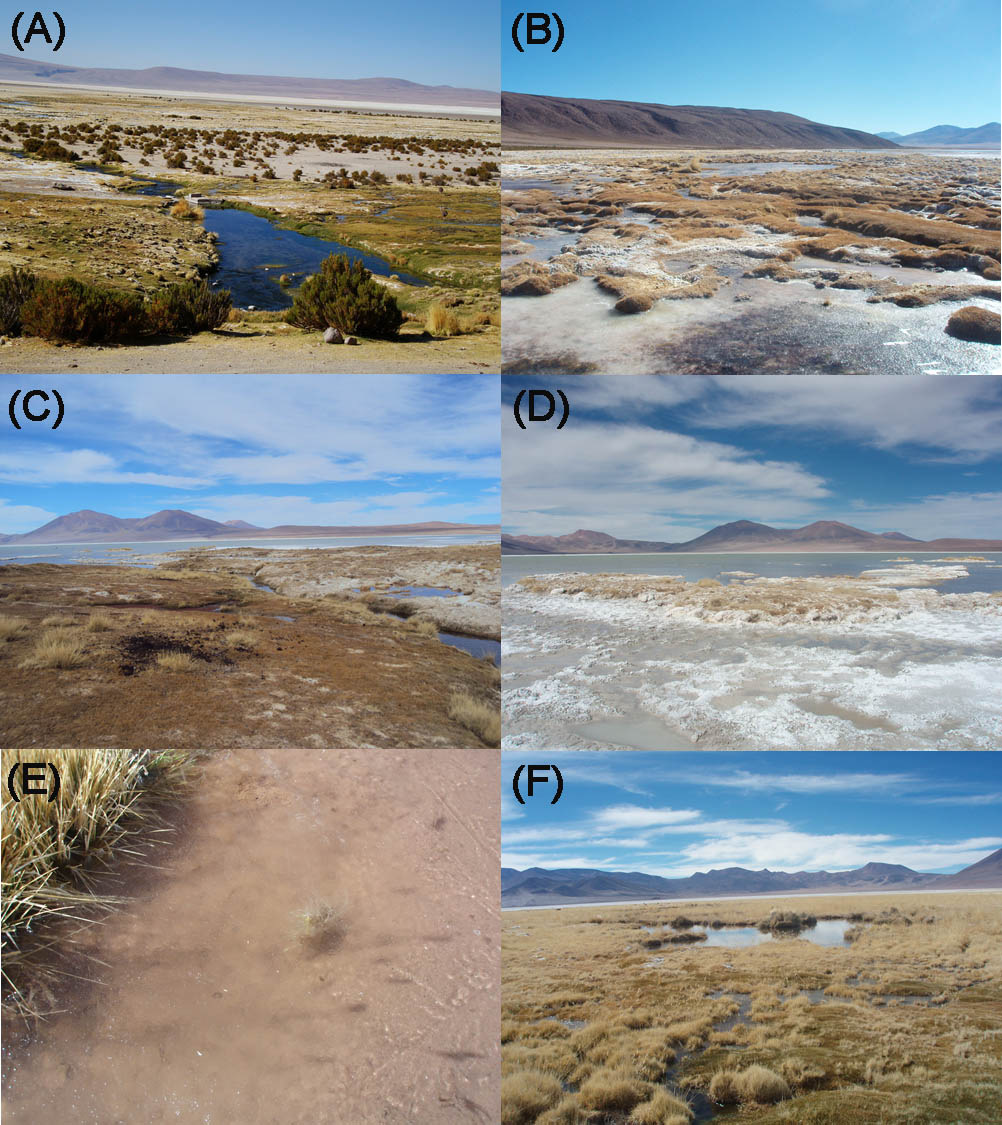


Figure S2. Sampling sites images for July 2012 (dry season). (A) and (B) H0 spring and pond sites, respectively (C) and (D) H3 and H4 sites landscapes, respectively, (E) and (F) H6 pond site showing microbial mats below the frozen surface layer and H6 landscape, respectively.


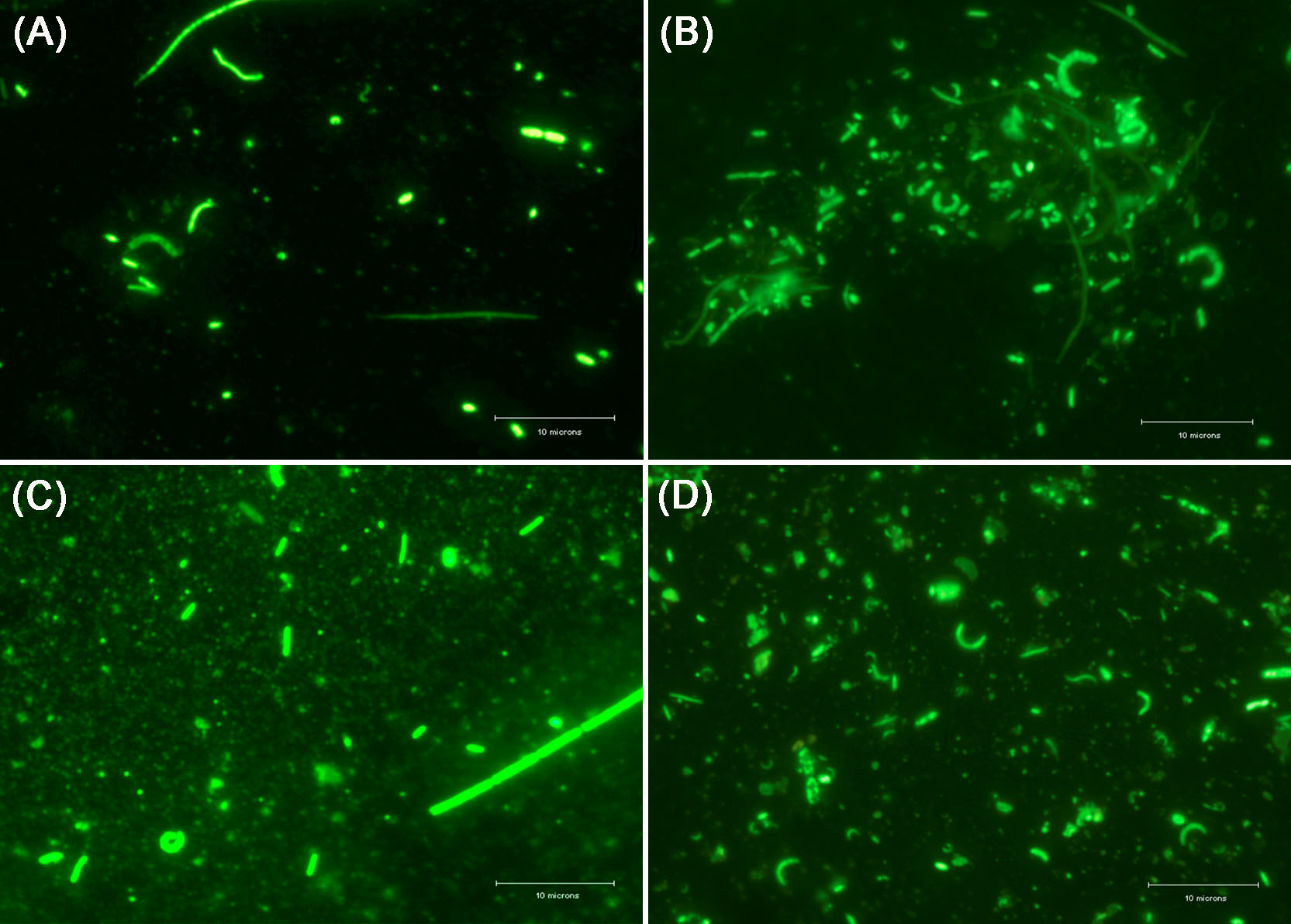


Figure S3. Images showing picoplankton and viruses during wet season, visualized with epifluorescence microscopy and stained with SYBR-Gold. Scale bar = 10 µm (A) and (B) H0 pond site showing free microbes and an aggregation, respectively, (C) and (D) H5 and H3 pond sites non diluted samples, respectively.


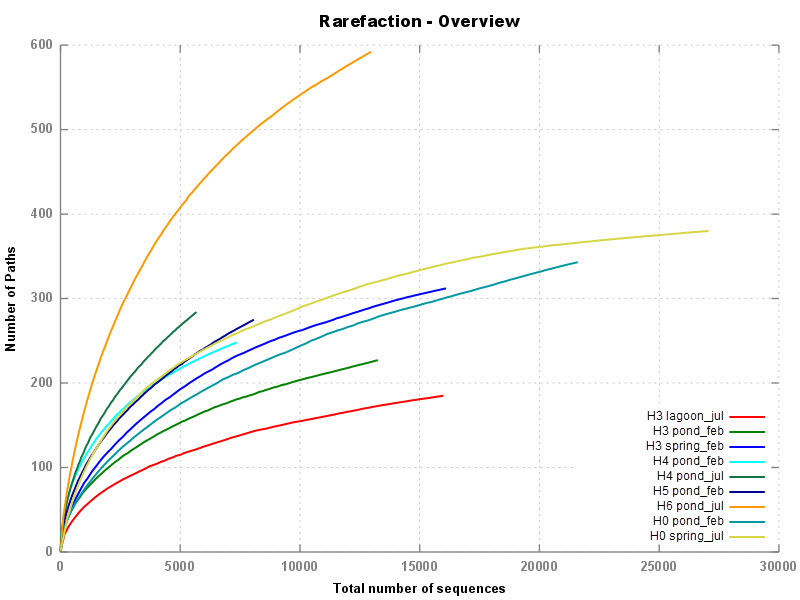


Figure S4. Rarefaction overview curves for the total number of sequences at the different sampling sites during contrasting seasons (wet versus dry).


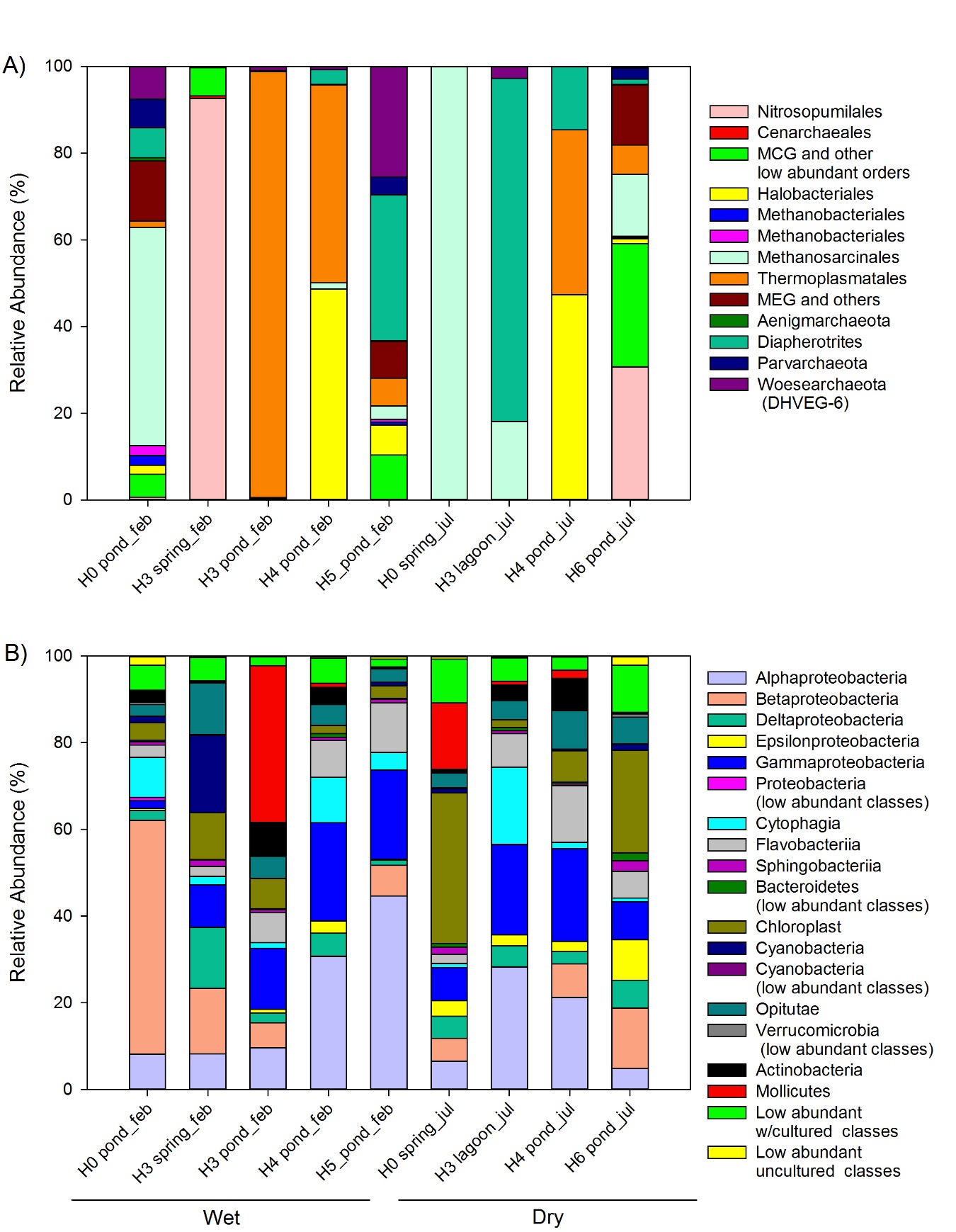


Figure S5. (A) and B) Archaeal and bacterial order and class relative contribution, respectively, retrieved at the different sampling sites during contrasting seasons (wet versus dry).


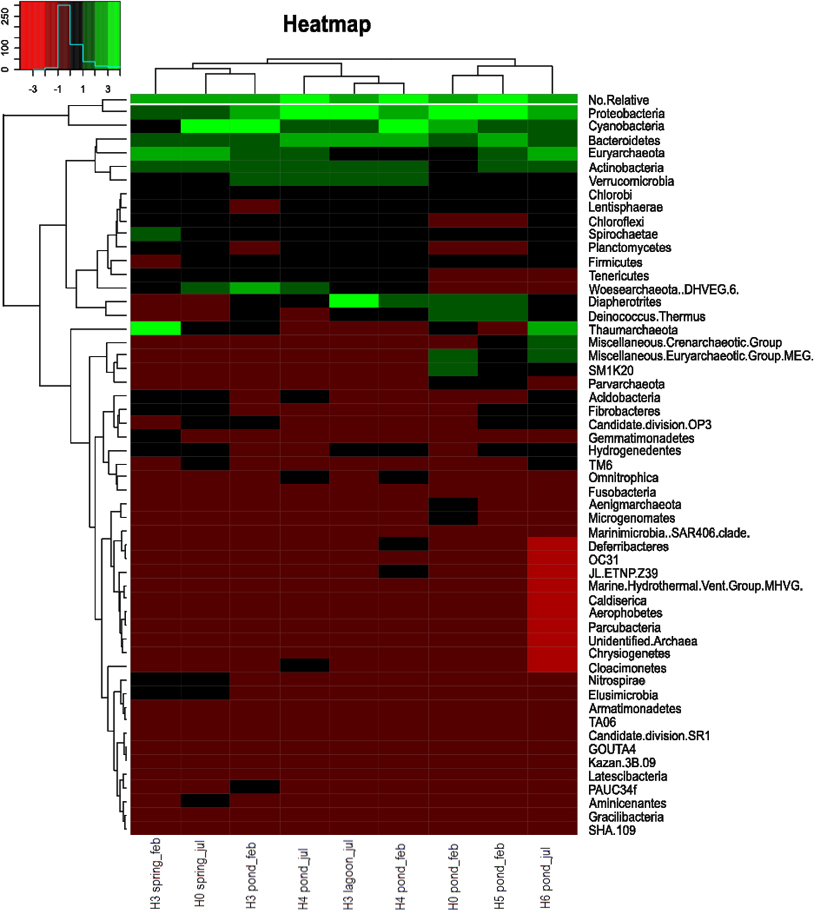


Figure S6. Cluster and heatmap analysis of the active microbial community structure for the different sampling sites.

Table S1. Physical and chemical parameters determined during sampling.

| Site | Date | Sampling  Time | Monthly Average  Precipitation | Temperature  (°C) | Conductivity  (µS cm^-1^) | Dissolved Oxygen  (mg L^-1^) | pH | NH_4_^+^  (µmol L^-1^)  +SD | NO_3_^-^  (µmol L^-1^)  +SD | | NO_2_^-^  (µmol L^-1^)  +SD | | PO_4_^3-^  (µmol L^-1^)  +SD |
| --- | --- | --- | --- | --- | --- | --- | --- | --- | --- | --- | --- | --- | --- |
|  |  |  | (mm)* |  |  |  |  |  | | | | | |
| H0 pond | 07-feb-12 | 9:00 | 75.2 | 14.5 | 457 | 7.70 | 7.70 | 0.30  +0.11 | 11.08** | 0.37  ** | | 0.85  ** | |
| H3 spring | 04-feb-12 | 13:00 |  | 16.5 | 434 | 4.62 | 8.15 | 0.27  +0.12 | 18.69+0.11 | 0.27  +0.05 | | 0.60  +0.04 | |
| H3 pond | 04-feb-12 | 12:00 |  | 22.5 | 1,300 | 7.70 | 8.99 | N.A. | N.A. | N.A. | | N.A. | |
| H4 pond | 04-feb-12 | 11:16 |  | 18.9 | 2,850 | 4.00 | 8.60 | 0.24  +0.19 | 0.45  +0.01 | 0.25  +0.02 | | 76.54  +0.72 | |
| H5 pond | 04-feb-12 | 8:46 |  | 11.3 | 1,400 | 6.00 | 9.30 | 0.60  +0.15 | 3.12  ** | 0.30  ** | | 0.91  ** | |
| H0 spring | 03-jul-12 | 15:00 | 0 | 14.6 | 476 | 5.80 | 8.35 | 0.83  +0.04 | 8.92  +0.04 | 0.41  +0.01 | | 5.43  +0.07 | |
| H3 lagoon | 03-jul-12 | 14:22 |  | 14.1 | 51,000 | 5.90 | 8.55 | 0.59  +0.15 | 2.31  +0.08 | 0.10  +0.02 | | 202.69  +5.84 | |
| H4 pond | 03-jul-12 | 12:35 |  | 5.1 | 37,000 | 8.50 | 8.47 | 0.38  +0.12 | 3.00  +0.32 | 0.21  +0.02 | | 105.29  +12.8 | |
| H6 pond | 03-jul-12 | 10:48 |  | 5.3 | 806 | 7.33 | 6.32 | 3.09  +0.38 | 5.72  ** | 0.68  ** | | 8.09  ** | |
|  | | | | | | | | | | | | | |

N.A. (Not Available), * data obtain from meteorological stations situated at Altos del Huasco hills limiting Salar de Huasco basin situated at 4,044 m.a.s.l., Latitude 20°10´13’’, Longitude 68°46’13’’, available online (http://snia.dga.cl/BNAConsultas/reportes from DGA, Chile).

+ Denotes standard deviation (SD) from replicates. ** SD derived from pseudo-replicates from autoanalyzer (NO_3_^-^, NO_2_^-^, PO_4_^3-^, were <0.58, 0.06, <0.05 µM, respectively).

Table S2. Viral and picoplankton abundance and virus to picoplankton ratio (VPR), determined by epifluorescence microscopy (EFM) and flow cytometer (FCM).

| Site | Date | Virus | Virus  ± SD | Picoplankton (FCM) | Picoplankton ±SD (FCM) | VPR | VPR ±SD | Picoplankton (EFM) | Picoplankton ±SD (EFM) | VPR  (EFM) |
| --- | --- | --- | --- | --- | --- | --- | --- | --- | --- | --- |
|  |  | [VLP mL^-1^] | [VLP mL^-1^] | [cells mL^-1^] | [cells mL^-1^] |  |  | [cells mL^-1^] | [cells mL^-1^] |  |
| H0 pond | 03-feb-12 | 6.73E+06 | 1.69E+08* | 1.00E+05 | 9.15E+05** | 67 | 151*** | N.A. | N.A. | - |
|  | 05-feb-12 | 1.29E+08 | ±1.78E+08 | N.A. | ±7.06E+05 | N.A. | ±172 | N.A. | N.A. | - |
|  | 06-feb-12 | 2.01E+08 |  | N.A. |  | N.A. |  | N.A. | N.A. | - |
|  | 06-feb-12 | 4.95E+07 |  | 1.33E+06 |  | 37 |  | 5.55E+04 | 7.16E+03 | 892 |
|  | 07-feb-12 | 4.59E+08 |  | 1.31E+06 |  | 349 |  | 1.21E+06 | 6.16E+02 | 380 |
| H3 spring | 04-feb-12 | 1.09E+07 | ±2.78E+05 | 6.93E+06 | N.A. | 2 | - | 3.27E+06 | 1.14E+05 | 3 |
| H4 pond | 04-feb-12 | 3.83E+08 | ±8.02E+07 | 1.34E+07 | N.A. | 29 | - | N.A. | N.A. | - |
| H5 pond | 04-feb-12 | 4.78E+08 | ±2.05E+07 | 1.36E+06 | N.A. | 351 | - | 1.35E+06 | 9.42E+04 | 354 |
| H0 spring | 03-jul-12 | 8.44E+05 | ±5.09E+05 | 1.07E+05 | N.A. | 8 | - | N.A. | N.A. | - |
| H3 lagoon | 03-jul-12 | 4.26E+08 | ±5.64E+07 | 1.83E+07 | N.A. | 23 | - | N.A. | N.A. | - |
| H4 pond | 03-jul-12 | 2.78E+08 | ±5.16E+06 | 4.50E+06 | N.A. | 62 | - | N.A. | N.A. | - |
| H6 pond | 03-jul-12 | 2.04E+07 | ±5.97E+06 | 1.08E+06 | N.A. | 19 | - | N.A. | N.A. | - |

N.A. (data not available)

*viruses mean value, H0 site, n=5, **picoplankton mean value, H0 site, n=3, ***VPR mean value, H0 site, n=3.

+ Denotes standard deviation (SD) that correspond to pseudo-replicates, excluding the ones that derived from samples taken at H0 pond (marked with asterisks).

Table S3: Spearman Rank Order Correlations. Marked correlations are significant at p < 0.05, n = 9 and 8 for physical and biochemical variables, respectively.

| Variable | Temp | Cond | O_2_ | pH | NH_4_^+^ | NO_3_^-^ | NO_2_^-^ | PO_4_^3-^ | Picoplankton | Virus |
| --- | --- | --- | --- | --- | --- | --- | --- | --- | --- | --- |
| Temperature | 1.00 |  |  |  |  |  |  |  |  |  |
| Conductivity | -0.32 | 1.00 |  |  |  |  |  |  |  |  |
| O_2_ | -0.47 | 0.13 | 1.00 |  |  |  |  |  |  |  |
| pH | 0.27 | 0.58 | -0.08 | 1.00 |  |  |  |  |  |  |
| NH_4_^+^ | -0.60 | 0.07 | 0.36 | -0.21 | 1.00 |  |  |  |  |  |
| NO_3_^-^ | 0.14 | -0.93 | 0.10 | -0.69 | 0.10 | 1.00 |  |  |  |  |
| NO_2_^-^ | -0.02 | -0.67 | 0.12 | -0.60 | 0.55 | 0.60 | 1.00 |  |  |  |
| PO_4_^3-^ | -0.36 | 0.93 | 0.12 | 0.36 | 0.14 | -0.86 | -0.55 | 1.00 |  |  |
| Picoplankton | 0.19 | 0.60 | -0.48 | 0.55 | -0.43 | -0.62 | -0.83 | 0.52 | 1.00 |  |
| Virus | -0.29 | 0.74 | -0.02 | 0.76 | -0.05 | -0.74 | -0.57 | 0.48 | 0.64 | 1.00 |

Picopankton: measured by FCM

Virus: measured by EFM

Table S4: SIMPER analysis, similarity percentages versus domains contributions, One-Way Analysis. Groups High and Low viral abundance (10^8^ VLP mL^-1^ as threshold). Average dissimilarity = 23.19.

| Domain | Av.Abund | Av.Abund | Av.Diss | Diss/SD | Contrib% | Cum.% |
| --- | --- | --- | --- | --- | --- | --- |
| Unclassified | 5.62 | 2.85 | 9.48 | 1.65 | 40.88 | 40.88 |
| Archaea | 4.66 | 7.50 | 9.06 | 1.46 | 39.06 | 79.94 |
| Bacteria | 6.31 | 5.62 | 4.65 | 1.67 | 20.06 | 100.00 |
